# Single-Cell Atlas Reveals the Hemocyte Subpopulations and Stress Responses in Asian Giant Softshell Turtle during Hibernation

**DOI:** 10.3390/biology12070994

**Published:** 2023-07-12

**Authors:** Xiaoyou Hong, Yakun Wang, Kaikuo Wang, Chengqing Wei, Wei Li, Lingyun Yu, Haoyang Xu, Junxian Zhu, Xinping Zhu, Xiaoli Liu

**Affiliations:** 1 Key Laboratory of Tropical and Subtropical Fishery Resources Application and Cultivation, Ministry of Agriculture and Rural Affairs, Pearl River Fisheries Research Institute, Chinese Academy of Fishery Sciences, Guangzhou 510380, China; hongxiaoyou1216@163.com (X.H.); wykzkyky@163.com (Y.W.); kaikuo_0321@163.com (K.W.); wcq1970@163.com (C.W.); liwei@prfri.ac.cn (W.L.); lysnp@163.com (L.Y.); 18457173557@163.com (H.X.); zhujunxian_1994@163.com (J.Z.); 2 College of Life Science and Fisheries, Shanghai Ocean University, Shanghai 201306, China

**Keywords:** single cell, transcriptome, hemocytes, hibernation, *Pelochelys cantorii*

## Abstract

**Simple Summary:**

Hibernation in turtle species is an adaptive survival strategy to colder winter conditions or food restrictions. However, the mechanisms underlying seasonal adaptions remain unclear. In this study, we isolated and performed scRNA-seq analysis on *P. cantorii* hemocytes in the active state and during hibernation to classify the hemocyte types and obtain the relevant cell maps. We identified the marker genes of each cell population and the differentiation pathways associated with their maturation. We also discovered different immune-related genes that might play crucial roles in hemocyte differentiation before and after hibernation. Our study aims to provide a reference dataset for the unified classification of *P. cantorii* hemocytes and improve our understanding of the immune system of *P. cantorii*.

**Abstract:**

Hibernation in turtle species is an adaptive survival strategy to colder winter conditions or food restrictions. However, the mechanisms underlying seasonal adaptions remain unclear. In the present study, we collected hemocytes from *Pelochelys cantorii* and compared the molecular signature of these cells between the active state and hibernation period based on single-cell RNA sequencing (scRNA-seq) analysis. We found six cell types and identified a list of new marker genes for each cell subpopulation. Moreover, several heat shock genes, including the Hsp40 family chaperone gene (*DNAJ*) and HSP temperature-responsive genes (*HSPs*), were upregulated during the hibernation period, which predicted these genes may play crucial roles in the stress response during hibernation. Additionally, compared to hemocytes in the active state, several upregulated differentially expressed immune-related genes, such as *stat1*, *traf3*, and *socs6*, were identified in hemocytes during the hibernation period, thus indicating the important immune function of hemocytes. Therefore, our findings provide a unified classification of *P*. *cantorii* hemocytes and identify the genes related to the stress response, thereby providing a better understanding of the adaptive mechanisms of hibernation.

## 1. Introduction

Hemocytes have significant roles in homeostasis maintenance and immunity and coagulation [1,2,3], and research on hemocytes can provide a better understanding of the relationship between their normal and pathological functions and animal growth and development, mature reproduction, living environment and other factors from the perspective of comparative hematology and comparative immunology [4,5,6,7]. Moreover, such research has also explored the trends and characteristics of hemocytes in phylogenetic evolution [8]. The earliest research on reptile hematology, including on the microscopic structure, number, and size of hemocytes, can be traced back to the 1940s [9,10,11]. However, few studies have been performed on the hemocytes of turtles, which is an ancient group of reptiles, and seven or eight types of hemocytes of turtles have been revealed based on electron microscopy [12,13].

The Asian giant softshell turtle (*Pelochelys cantorii*) belongs to the family Trionychidae and is distributed in Southeast Asia, including China, India, Bangladesh, Myanmar, Laos, Thailand, Cambodia, Vietnam, Malaysia, and Indonesia. *P. cantorii* has been listed as a first-class national protected animal in China since 1989 due to overhunting and habitat destruction [14,15,16]. The early life morphology of *P. cantorii* is similar to that of the Chinese soft-shell turtle (*Pelodiscus sinensis*); thus, *P. cantorii* is not always identified as a separate species, is confused with *P*. *sinensis*, and is eaten illegally [17]. Moreover, *P. cantorii* is easy to spot because of its large size, and its long period of sexual maturation has also accelerated its extinction. A recent survey showed that only 13 wild *P. cantorii* were kept in captivity in 6 different locations [14]. Hence, it is important and interesting to classify the cell types present in hemocytes to provide a deeper understanding of the immune system of *P. cantorii*.

Single-cell RNA sequencing (scRNA-seq) is a new technology for high-throughput sequencing analyses of genomes, transcriptomes, and epigenomes at the level of a single cell. ScRNA-seq can annotate unclassified cells according to the mRNA expression pattern of each cell and, thus, is a particularly powerful tool for analyzing cell diversity and heterogeneity, predicting the functions of single-cell populations, and determining functional roadmaps of cell differentiation [18,19,20]. Single-cell molecular profiling is widely expected to make important contributions to our understanding of many fields, such as neurobiology [21], organ growth [22], cancer biology [23], clinical diagnosis [24], immunology [25], microbiology [26] and embryology [27], and is becoming the new focus of life science research. In 2013, scRNA-seq was rated as the annual technology by Nature Methods [28]. In the same year, Science Magazine ranked scRNA-seq as the top technology among the six most noteworthy fields and suggested that this technology would change many fields of biology and medicine [29]. Currently, the use of scRNA-seq for the hemocytes of animals, especially in mammals, has exploded, and these large-scale datasets range from identifying previously well-defined hemocyte populations [20,30,31] to covering the whole organization [32,33]. However, although extensive studies have been performed on mammals or other aquatic animals, no reports have been published on the hemocytes of *P. cantorii* based on the single-cell level.

Here, we isolated and performed scRNA-seq analysis on *P. cantorii* hemocytes in the active state and during hibernation to classify the hemocyte types and obtain the relevant cell maps. We identified the marker genes of each cell population and the differentiation pathways associated with their maturation. We also discovered different immune-related genes that might play crucial roles in hemocyte differentiation before and after hibernation. Our findings provide a reference dataset for the unified classification of *P. cantorii* hemocytes and improve our understanding of the immune system of *P. cantorii*.

## 2. Materials and Methods

### 2.1. Cell Preparation of Single Hemocytes in Suspension

*P. cantorii* specimens were acquired from the Asian giant softshell turtle artificial breeding base of the Ministry of Agriculture and Rural Affairs, Foshan, Guangdong, China. The mean weight of *P. cantorii* was 650.82 g during the active phase of the year (summer 2022) and 666.57 kg during the hibernation period (January 2022). Individuals during the active phase were cultured in outdoor cement ponds with circulating filtered water. The water depth in the pool was 50 cm, and the sand thickness at the bottom of the pool was 30 cm. The cultured density was 1 animal per square meter. When the water temperature drops to about 10 °C degrees in January, put *P. cantorii* in a box with water in the incubator. The water depth in the box was just above the turtle’s back. Then, we reduced the temperature by 1 degree every 2 days and kept it at 4 °C for 2 weeks before sampling. At this time, the turtles were in the maintenance period of hibernation. To reduce the chance brought by individual differences, 5 mL blood of 5 *P. cantorii* specimens in the active state and 5 mL blood of these identical 5 *P. cantorii* specimens during the hibernation period were collected from the jugular vein using an anticoagulant solution, respectively. There were two biological repeats in the active state and hibernation period of individuals. The blood and lymphocyte separation solution was placed in a 20 °C water bath for 20 min, and the whole blood was diluted with the same amount of Hank’s balanced salt solution (HBSS). Subsequently, 4 mL whole blood was slowly added to 2 mL lymphocyte separation solution so that the amount of diluted blood was superplaced on the stratified solution to keep the interface clear, and the solution was then centrifuged at 500× *g* for 20 min. After discarding the top layer, the mononuclear cells of the white membrane layer were absorbed along the periphery of the centrifuge tube wall and transferred to another centrifuge tube. Then, 5 mL of HBSS was added for washing and centrifugation at 300× *g* for 10 min, and the supernatant was discarded. The hemocyte pellet was resuspended using 1 mL of HBSS. Cell number and concentration were assessed by Countess^®^ II Automated Cell Counter, and the cell concentration was adjusted to the ideal concentration, which was generally not less than 1000 cells/μL. All animal experiments in this research were performed according to the guidelines established by the Pearl River Fisheries Research Institute, the Chinese Academy of Fishery Sciences. The turtles used were treated humanely and ethically, and the experiments were approved by the Laboratory Animal Ethics Committee of the Pearl River Fisheries Research Institute, Chinese Academy of Fishery Sciences.

### 2.2. Library Construction and Single-Cell RNA Sequencing

Library synthesis and single-cell RNA sequencing were performed by Gene Denovo (Guangzhou, China). In detail, single hemocytes were barcode-labeled and mixed with reverse transcriptase, and then wrapped by oil surfactant droplets located in the microfluidic “double cross” system to form gel beads-in-emulsions (GEMs). GEMs flowed into the reservoir and were collected. Then, silane magnetic beads were used to remove the remaining biochemical reagents and primers from the post-GEM reaction mixture. Subsequently, full-length, barcoded cDNAs were amplified by PCR with the sequencing primer R1 and P5 arms and subsequently sequenced on the Illumina 10×Genomics Chromium platform (Illumina Novaseq 6000).

### 2.3. Data Quality Control and Gene Expression Quantification

Next, 10× Genomics Cell Ranger software (http://support.10xgenomics.com/single-cell/software/overview/welcome, accessed on 12 November 2021) was used to convert raw BCL files to FASTQ files and then perform alignment and count quantification. Briefly, reads with low-quality barcodes and UMIs were filtered out and then mapped to the reference genome. Reads uniquely mapped to the transcriptome and intersecting an exon at least 50% were considered for UMI counting. Before quantification, the UMI sequences were corrected for sequencing errors, and valid barcodes were identified based on the EmptyDrops method. The cell-by-gene matrices were produced via UMI counting and cell barcode calling.

### 2.4. Cell Clustering

After removing low-quality cells, we employed the global-scaling normalization method, “LogNormalize”, which normalizes the gene expression measurements for each cell by the total expression, multiplies the measurements by a scale factor (10,000 by default), and log-transforms the results. The formula is shown as follows: gene expression level = log (1 + (UMIA ÷ UMITotal) × 10,000). The normalized expression matrix is then scaled and subjected to principal component analysis (PCA) for dimensional reduction. A resampling test inspired by the JackStraw procedure is then performed [34]. We randomly permuted a subset of the data (1% by default) and rerun PCA, constructing a ‘null distribution’ of gene scores, and repeated this procedure. We identified ‘significant’ PCs as those with a strong enrichment of low p value genes for downstream clustering and dimensional reduction. 

Seurat software implements a graph-based clustering approach. Distances between the cells were calculated based on previously identified PCs. Briefly, Seurat embeds cells in a shared nearest-neighbor (SNN) graph, with edges drawn between cells via similar gene expression patterns. To partition this graph into highly interconnected quasi-cliques or communities, we first constructed the SNN graph based on the Euclidean distance in PCA space and refined the edge weights between any two cells based on the shared overlap in their local neighborhoods (Jaccard distance). We then cluster cells using the Louvain [35] method to maximize modularity. For the visualization of clusters, t-distributed stochastic neighbor embedding (t-SNE) was generated using the same PCs [36]. 

### 2.5. Differentially Expressed Genes (DEGs) between P. cantorii in the Active State (AS) and during the Hibernation (HI) Period

To further analyze the characteristics of transcriptional regulation patterns of each cell subpopulation and screen the marker genes specifically expressed from each subpopulation, the expression value of each gene in the given cluster was compared against the rest of the cells using the Wilcoxon rank sum test in Seurat software [37]. Significant upregulated genes were identified using a number of criteria: genes had to be at least 1.28 fold overexpressed in the target cluster; genes had to be expressed in more than 25% of the cells belonging to the target cluster; p value is less than 0.05.

Moreover, genes usually interact with each other to play roles in certain biological functions. Pathway-based analysis helps to further understand gene biological functions, and KOG is the major public pathway-related database [38]. Pathway enrichment analysis identified significantly enriched metabolic pathways or signal transduction pathways in differentially expressed genes compared with the whole genome background. The calculated p value was subjected to FDR correction, taking FDR ≤ 0.05 as a threshold. Pathways meeting this condition were defined as significantly enriched pathways in differentially expressed genes.

### 2.6. Prediction of Cell Cycles and Analysis of Cell Trajectory

To calculate the cell cycle, the Seurat AddModuleScore function was used to score the possible cell cycle period based on the expression of characteristic cycle genes [39]. Cells with a high score of less than 0.3 were identified as noncycling cells [40]. Single-cell pseudotime trajectories were performed using Monocle 3 [41] (https://github.com/cole-trapnell-lab/monocle3, accessed on 14 February 2023). The RNA velocity analysis was performed using the package velocyto with the default parameters, and the BAM files were used as inputs. For visualization, velocity vectors were plotted as locally average vector fields on the tSNE embeddings of our high-quality cells from the previous step [42].

## 3. Results

### 3.1. Global Transcriptome Profile of Hemocytes of P. cantorii

**Figure 1:**
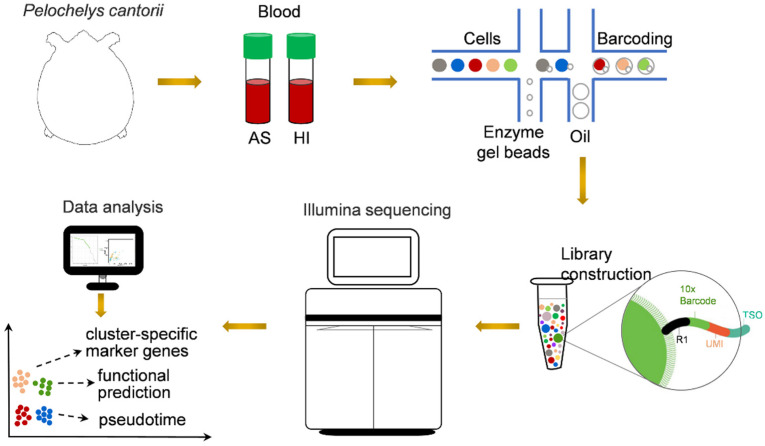
Schematic representation of scRNA-seq analysis of *P. cantorii* hemocytes. AS and HI represent hemocytes of active state and hibernation period, respectively.

To obtain comprehensive information on hemocytes, we collected blood from *P. cantorii* in the active state and during the hibernation period. The samples were then prepared for 3′ scRNA-seq using the 10× Genomics platform (Figure 1). After sequencing and standard quality control, a total of 13,552, 11,527, 2367, and 24,727 cells were generated in hemocytes in the active state and during the hibernation period, respectively (Table 1). An average of 90.80% (active state) and 93.00% (hibernation period) of the clean reads were confidently mapped to the *P. cantorii* genome (Table 1). We also detected a median of 763 genes and 1652 unique molecular identifier (UMI) counts per cell in hemocytes of the active state and a median of 827 genes and 2507 UMI counts per cell in hemocytes of the hibernation period (Table 1). Moreover, the proportions of Q30 bases in the UMI of hemocytes in the active state and during the hibernation period were 94.50% and 96.50%, respectively. Taken together, the assembly produced a high-quality transcriptional spectrum, which was appropriated for our subsequent analyses.

### 3.2. Identification of Cell Subtypes of P. cantorii Hemocytes

**Figure 2:**
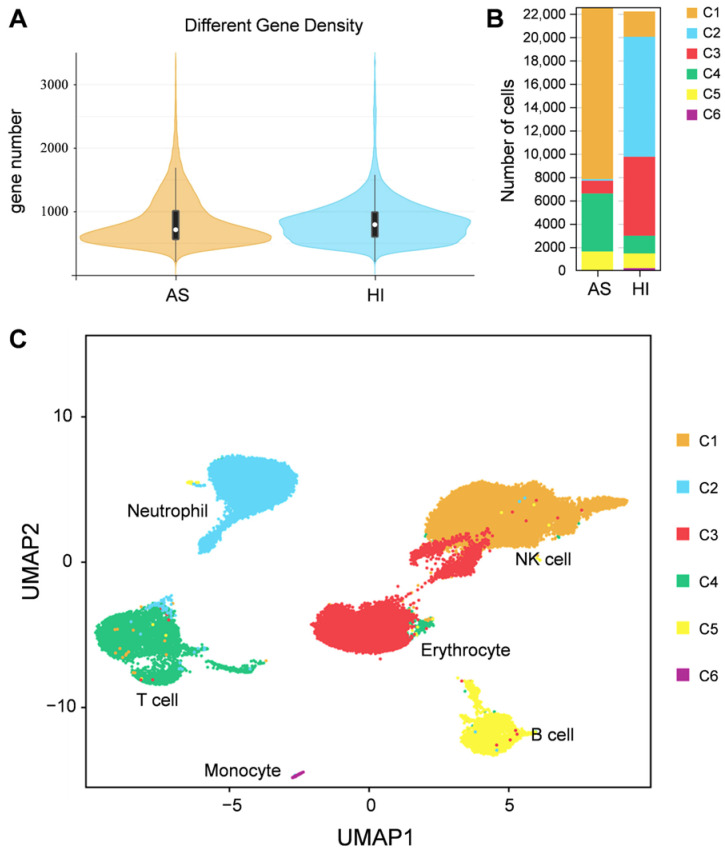
Single-cell profiling of cell populations in *P. cantorii* hemocytes. (**A**) Distribution map of basic cell information. The violin column represents the distribution status of measured gene number of each sample. (**B**) Percentage of cells in each cluster and their proportional distribution in the total hemocyte dataset. (**C**) Visualization of major cell types UMAP of *P. cantorii* hemocytes. Different cell types are shown in distinct colors.

**Figure 3:**
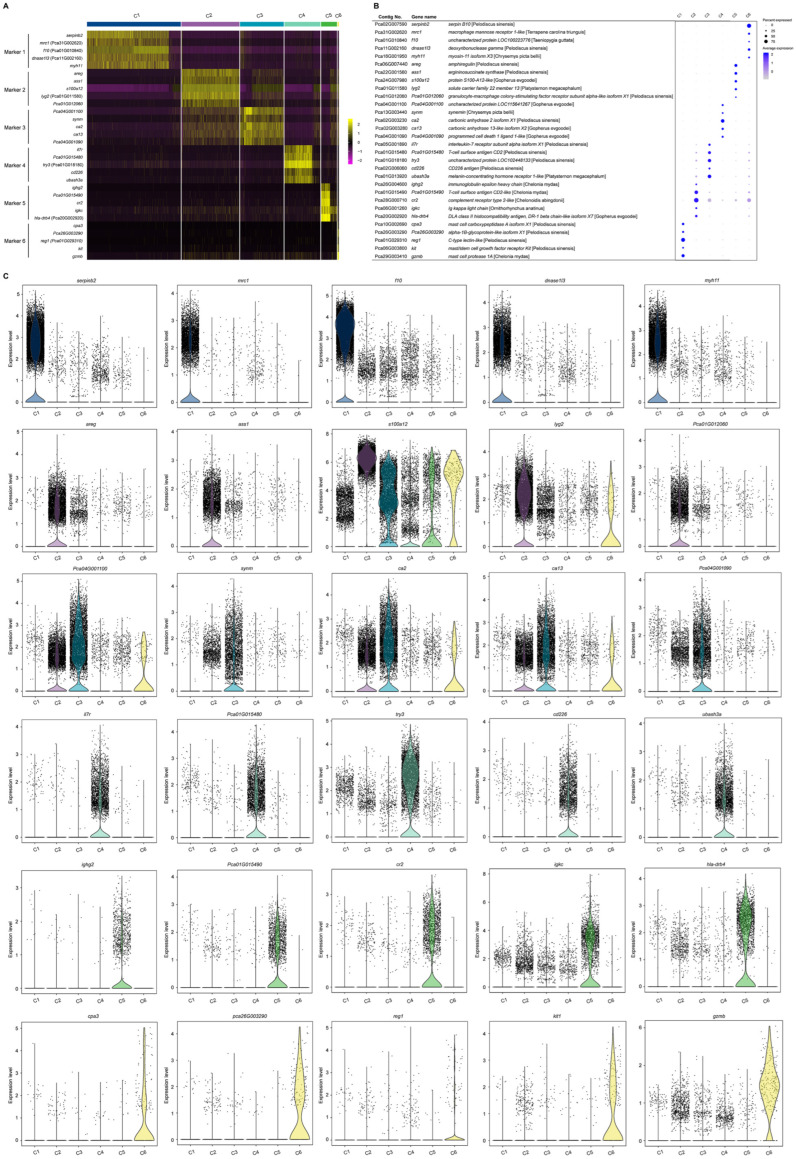
The cluster-specific marker genes of *P. cantorii* hemocytes predicted using the Seurat. (**A**) Heatmap profile of the marker in each cluster. Color gradient from red to yellow represents the expression level from low to high of each single cell. (**B**) Important marker genes in each cluster. Color gradient of the dot represents the expression level of each gene, and the size represents the percentage of cells expressing any genes per cluster. (**C**) Violin plots of the normalized expression of important marker genes in the nine clusters.

To group single cells with similar gene expression, filtration of the abnormal cells is necessary, including the removal of polycells based on the cellular rate and elimination of abnormal cells based on the gene and UMI number. DoubletFinder (v2.0.3) [43] was used to calculate the probability of GEM multicellular (proportion of artificial k-nearest neighbors), and then the multicellular rate of each sample was calculated based on the relationship between the effective cell number given by 10X official and the multicellular rate, and the multicellular filtering threshold of each sample was determined (NS: 9.55; HI: 1.79). Additionally, cells detected with less than 200 genes or more than 1600 genes and UMIs numbering more than 27,000 were excluded. After filtration, we obtained a total of 11,262 cells and a median value of 1488 UMIs and 723 genes per cell in hemocytes of the active state and a total of 11,104 cells and a median value of 2251 UMIs and 765 genes per cell in hemocytes of the hibernation period (Figure 2A and Figure S1, and Table S1). Based on the mutual nearest neighbor methodology, we first combined the active state samples and hibernation period and found there are 6 cell types in *P. cantorii* hemocytes (Figure 2B,C). Moreover, we found that clusters 1, 4, and 3 showed a high proportion of cells in hemocytes of the active state, while cells in clusters 2, 3, and 1 were highly expressed in the hibernation period (Figure 2B and Table S2). A total of 1701 markers were then predicted in hemocytes (Table S3). For each cluster, 433 (Cluster 1), 349 (Cluster 2), 90 (Cluster 3), 459 (Cluster 4), 280 (Cluster 5), and 120 (Cluster 6) markers were identified (Table S3). We then showed the top 5 expressed genes in each cluster of hemocytes and the heatmap results revealed that PCA-based cell separation was desirable in our analysis because a clear separation boundary for each cluster was detected (Figure 3A). Dot plots (Figure 3B) and violin plots (Figure 3C) of the normalized expression profiles of genes in each cell type were consistent with the cell classification displayed in the heatmap results (Figure 3A), suggesting that these genes from the nine cell groups were classically representative and well clustered. 

To further characterize the clusters, we inferred the cell populations based on known markers and a single-cell database of chicken, human, and red-eared slider turtle samples. Cluster 1, with the expression of the natural killer (NK) cell-related genes *cd9*, *cd151*, *itga2b*, and *actb* was considered the NK-cell population (Table S3). The neutrophil (cluster 2) population was determined by *lyz*, *mmp9*, and *cd99l2*. Cluster 3, the erythrocyte population, was confirmed by *alas2*, *hbb*, *hbaa*, and *gata1* (Table S3). Cluster 4, which expressed *ccr7* and other T-cell related genes (*tox*, *cd3e*, and *cd3g*), was clustered into a T-cell population, and Cluster 5, which expressed *cd74*, *cd79a*, *swap70*, *crip1*, *rpl21*, *rps27*, and *rps29* belonged to B-cell population (Table S3). In Cluster 6, the monocyte population was determined by *HLA-DRA* and *nfil3*. Thus, we applied a comprehensive investigation of *P. cantorii* hemocytes and successfully divided all the clusters into 6 cell populations including NK cells, erythrocytes, T cells, B cells, neutrophils, and monocytes (Figure 3).

### 3.3. DEG Analysis in Cell Clusters between Hemocytes of P. cantorii in Active State and Hibernation Period

**Figure 4:**
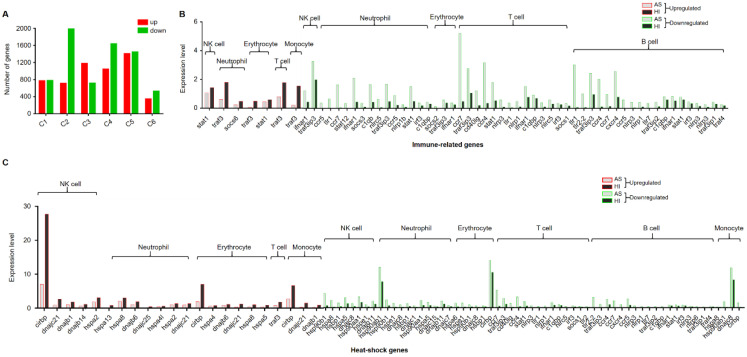
Differentially expressed genes (DEGs) in each cluster between cell clusters of *P. cantorii* hemocytes of active state and hibernation period. (**A**) Number of DEGs in each cluster. Red and green represent upregulated and downregulated genes, respectively. (**B**) The statistics of immune-related genes. Red and green borders represent upregulated and downregulated genes, respectively. Grey and black represent samples of active state and hibernation period, respectively. (**C**) The statistics of heat-shock genes. Red and green borders represent upregulated and downregulated genes, respectively. Grey and black represent samples of active state and hibernation period, respectively.

**Figure 5:**
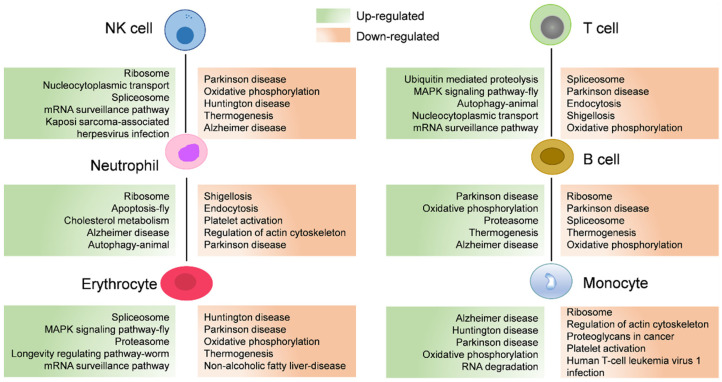
The associated enriched KEGG pathways of differentially expressed genes (DEGs) between cell populations of *P. cantorii* hemocytes of active state and hibernation period. Upregulated and downregulated pathways are annotated in green and orange boxes, respectively.

To investigate the responses to seasonal variation, we calculated the DEGs between cell populations of the hemocytes in the active state and during the hibernation period. A total of 12,546 DEGs were found with 5457 and 7089 significantly upregulated and downregulated genes, respectively (Figure 4A). Hemocytes play key roles in immunity [44]; here, we also identified immune-related genes from these DEGs and then analyzed their expression levels. Compared to hemocytes in the active state, most of these differentially expressed immune-related genes were downregulated in hemocytes during the hibernation period, except *stat1*, *traf3*, and *socs6*. As shown in Figure 4B, *stat1* was upregulated in NK cells and erythrocytes, *traf3* was upregulated in neutrophil cells, erythrocytes, T cells, and monocyte populations, and *socs6* was upregulated in neutrophil populations. Moreover, we also identified 62 differentially expressed heat-shock genes (Figure 4C). Among these DEGs, several genes were upregulated including *cirbp* and *traf3*, Hsp40 family chaperone genes (*DNAJ*) such as *dnajb1*, *dnajb6*, *dnajb14*, *dnajc21* and *dnajc25*, and HSP temperature-responsive genes (*HSPs*) such as *hspa2*, *hspa4*, *hspa4l*, *hspa5*, *hspa8* and *hspa13* (Figure 4C). Additionally, to further explore the pathways associated with DEGs, we performed a KOG function analysis of up- and downregulated DEGs. In the top 5 pathways, the upregulated genes were significantly enriched in the pathways such as “proteasome” and “nucleocytoplasmic transport” (Figure 5). Additionally, we found that several disease-associated pathways such as “endocytosis”, “platelet activation”, and “regulation of actin cytoskeleton” were abundant among downregulated DEGs (Figure 5). 

### 3.4. G2/M and S Phase Clustering and Pseudotime Trajectories of P. cantorii Hemocyte Clusters

**Figure 6:**
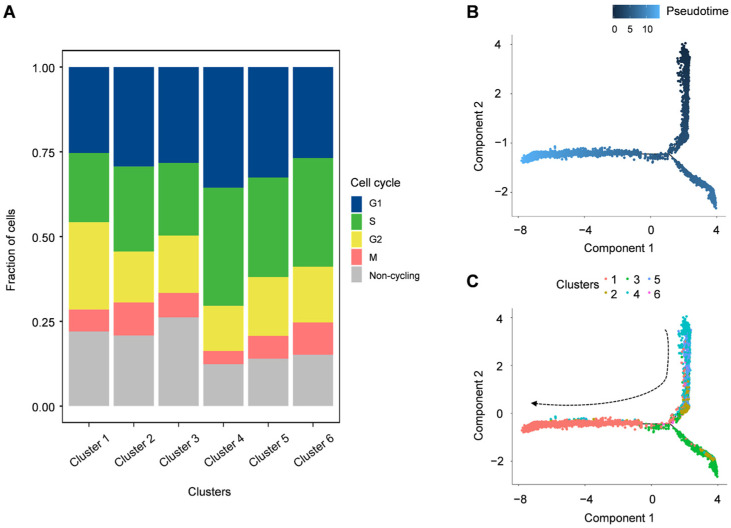
Cell cycle distribution of each cluster and pseudo-temporal ordering of hemocyte lineages. (**A**) Uniform manifold approximation and projection plot of cell cycles of *P. cantorii* hemocytes. (**B**) The degree of differentiation of the cell types of *P. cantorii* hemocytes in the pseudotime trajectory. Color gradient of each dot represents the pseudotime. (**C**) Developmental pseudotime trajectory of active state *P. cantorii* hemocytes. The colors indicate the various states of differentiation.

The cell cycle of each cell was calculated by the Seurat AddModuleScore function based on the expression of cycle marker genes in each stage. We found that all these sic clusters showed a high percentage in the G1/S phase (Figure 6A). For example, *ccne1* and *ccne2* are the main regulators of the transition from the G1 to S phase determining cell division, especially as putative genes associated with gene amplifications in various malignancies [45]. These findings suggested that these six hemocyte groups are tightly regulated by these G1/S-related genes to promote cell proliferation (Figure 6A). Furthermore, we performed lineage tree reconstruction using the Monocle 3 learn_graph function to investigate the differentiation dynamics of hemocytes. We considered Cluster 4 to be the initial state of hemocytes and set it as the starting point in the differentiation process because Cluster 4 was highly expressed bipotent progenitor or stem-like cell signatures *zeb1* and *itga6* (Figure 6B,C and Figure S2, Table S4). Our pseudo-temporal ordering analysis revealed that the hemocytes of *P. cantorii* differentiate from a single subpopulation into two major populations. Each cell type was pseudotime-distributed but not absolutely distributed on a specific branch, which indicated that the progenitors of each cell type had a physiological continuum from non-dividing to potentially dividing states (Figure S2).

## 4. Discussion

Hemocytes have been revealed to play crucial roles in nutrient uptake and storage [46], body homeostasis [47], and cellular and humoral immunity [48]. Identifying the single-cell transcriptome of hemocytes will provide a detailed and advanced understanding of cell classification. Although hemocytes have been well-studied in various groups ranging from humans to fishes [49,50,51,52], the type and function of hemocytes are still little known in reptiles. In this study, we performed comprehensive profiling of gene expression at the single-cell level in *P. cantorii* hemocytes. We first distinguished the classification of cell populations by sets of class-specific genes that are related to the specific function of each class of cells. Since recognized marker genes of *P. cantorii* hemocytes are not available, accurately classifying *P. cantorii* hemocytes is difficult. Marker genes provide a basis for identifying cell clusters; thus, here, we assigned the different clusters according to the expression characteristics and marker genes of humans or fishes and revealed nine and six subpopulations in hemocytes in the active state and during the hibernation period, respectively (Figure 2). The expression patterns of several marker genes differed among the multiple hemocyte clusters, suggesting that *P. cantorii* hemocytes were heterogeneous (Table S3). Moreover, an important contribution of this study was the identification of new marker genes in each hemocyte cluster, which may provide new insights into the characteristics of the transcriptional regulation patterns of hemocytes and the functional specialization of the cells (Figure 3 and Table S3). However, our classification of *P. cantorii* hemocytes cannot yet be regarded as conclusive results because several cell types, such as granulocytes, were not detected, which may be due to the absence of specific marker genes or because the cell counts were too low to be detected. Therefore, further studies combining morphological descriptions and single-cell sorting could be used to identify a more accurate and comprehensive classification of *P. cantorii* hemocyte populations. 

Hibernation is an adaptive survival strategy to colder winter conditions or food restrictions, and it occurs in many species, including reptiles [53], mammals [54], amphibians [55], and birds [56]. The physiological functions involved in minimizing energy expenditure, such as body temperature, heart rate, metabolic rate, and sensitivity to external stimuli, were drastically reduced during hibernation [57]. The hibernation period of *P. cantorii* usually starts in the middle of November and ends in the middle of April. Here, we found that the effective cell number of *P. cantorii* in the hibernation period was lower than that of the active state (Figure 2B), and this finding was different from that of hibernating *Helix pomatia*, in which neither temperature nor heart frequency seemed to influence the number of circulating cells [58]. Moreover, compared to hemocytes in the active state, only three differentially expressed immune-related genes, *stat1*, *traf3*, and *scos6*, were upregulated, while more genes belonged to NK cells, T cells, neutrophils, erythrocytes, and B cells were downregulated (Figure 4B). This result indicated that these genes may also be an important immune component in *P. cantorii* during hibernation. 

In addition, we also found that many genes encoding heat shock genes related to the stress response including Hsp40 family chaperone genes (*DNAJ*) and HSP temperature-responsive genes (*HSPs*) were differentially expressed between hemocytes in the active state and during the hibernation period (Figure 4C), which provided molecular evidence for the hemocyte protective mechanism under various stresses, such as thermal stress during hibernation. The upregulation of these genes, including *cirbp*, *traf3*, *dnajb1*, *dnajb6*, *dnajb14*, *dnajc21*, *dnajc25*, *hspa2*, *hspa4*, *hspa4l*, *hspa5*, *hspa8* and *hspa13*, may play a critical role during cell stress to prevent the appearance of folding intermediates that lead to misfolded or otherwise damaged molecules [59]. 

Moreover, most of the pathways significantly annotated by upregulated genes were enriched in “proteasome” and “nucleocytoplasmic transport” pathways (Figure 5), indicating enhanced protein synthesis and transport in the hemocytes of hibernating *P. cantorii*. This may be a response to protein damage caused by stress during hibernation. We also found that several metabolism-associated pathways such as “endocytosis”, “platelet activation”, and “regulation of actin cytoskeleton” were abundant among the downregulated DEGs (Figure 5). When the *P. cantorii* stays in the hibernating refuge, the expression of genes involved in metabolism-associated pathways were suppressed to save energy.

## 5. Conclusions

In this study, we isolated and performed scRNA-seq analysis on *P. cantorii* hemocytes in the active state and during hibernation to classify the hemocyte types and obtain the relevant cell maps. Our study aims to provide a reference dataset for the unified classification of *P. cantorii* hemocytes and improve our understanding of the immune system of *P. cantorii*.

## Figures and Tables

**Table 1:** Sequencing data of each sample.

Sample	Number of Reads	Valid Barcodes	Sequencing Saturation	Q30 Bases in Barcode	Q30 Bases in UMI	Estimated Number of Cells	Fraction Reads in Cells	Median Genes per Cell	Total Genes Detected	Median UMI Counts per Cell	Reads Mapped Confidently to Genome
AS-1	376,999,821	97.9%	80.6%	95.9%	92.4%	13,552	88.7%	634	15,573	1232	90.9%
AS-2	452,889,235	97.2%	83.6%	97.1%	96.6%	11,527	86.1%	891	16,425	2071	90.7%
HI-1	503,290,649	98.4%	91.9%	95.3%	94.7%	2367	58.2%	784	14,014	2080	94.4%
HI-2	400,484,177	96.4%	55.5%	96.9%	96.5%	24,727	81.8%	869	16,880	2934	91.6%

## Data Availability

Single-cell transcriptome raw data of *P. cantorii* were deposited in the NCBI Sequence Read Archive database under the BioSample accession number SRR20689449 (BioProject ID: PRJNA863082) (https://www.ncbi.nlm.nih.gov/bioproject/PRJNA863082, accessed on 28 July 2022).

## Supplementary Materials

The following supporting information can be downloaded at: https://www.mdpi.com/article/10.3390/biology12070994/s1. Figure S1: information on scRNA-seq data quality control. (A) Cell number of *P. cantorii* hemocytes before quality control. (B) Count number of *P. cantorii* hemocytes before quality control. (C) Cell number of *P. cantorii* hemocytes after quality control. (D) Count number of *P. cantorii* hemocytes after quality control. AS and HI represent hemocytes of active state and hibernation period, respectively. Figure S2: the subclusters of *P. cantorii* hemocytes distributed over the pseudotime differentiation trajectory. Table S1: statistical analysis of cell data in each sample before and after filtration. Table S2: statistical table of classification results of cell subsets. Table S3: the cluster-specific marker genes predicted in *P. cantorii* hemocytes. Table S4: the expression level of bipotent progenitor or stem-like cell signatures *zeb1* and *itga6* in hemocytes of an active state.
