# Supplementary material for: Single-Cell Atlas Reveals the Hemocyte Subpopulations and Stress Responses in Asian Giant Softshell Turtle during Hibernation
Source: Biology (Basel). 2023 Jul 12;12(7):994. doi: 10.3390/biology12070994 (PMC10376416; doi:10.3390/biology12070994)
Supplement: Supplementary file 1 [file biology-12-00994-s001.zip › biology-2403045-supplementary.pdf]

Single-cell atlas reveals the hemocyte subpopulations and stress responses in Asian giant softshell turtle during hibernation

Xiaoyou Hong[1], Yakun Wang[1], Kaikuo Wang[1, 2], Chengqing Wei[1], Wei Li[1], Lingyun Yu[1], Haoyang Xu[1, 2], Junxian Zhu[1, 2], Xinping Zhu[1, 2*], Xiaoli Liu[1*]

[1]Key Laboratory of Tropical and Subtropical Fishery Resources Application and Cultivation, Ministry of Agriculture and Rural Affairs, Pearl River Fisheries Research Institute, Chinese Academy of Fishery Sciences, Guangzhou 51038, China

[2] College of Life Science and Fisheries, Shanghai Ocean University, Shanghai 201306, China,

***Correspondence:**

Dr. Xiaoli Liu, liu_xiaoli1988@126.com, +86-020-81174522

Dr. Xinping Zhu, zhuxinping_1964@163.com, +86-020-81537378

Supplementary figures and tables

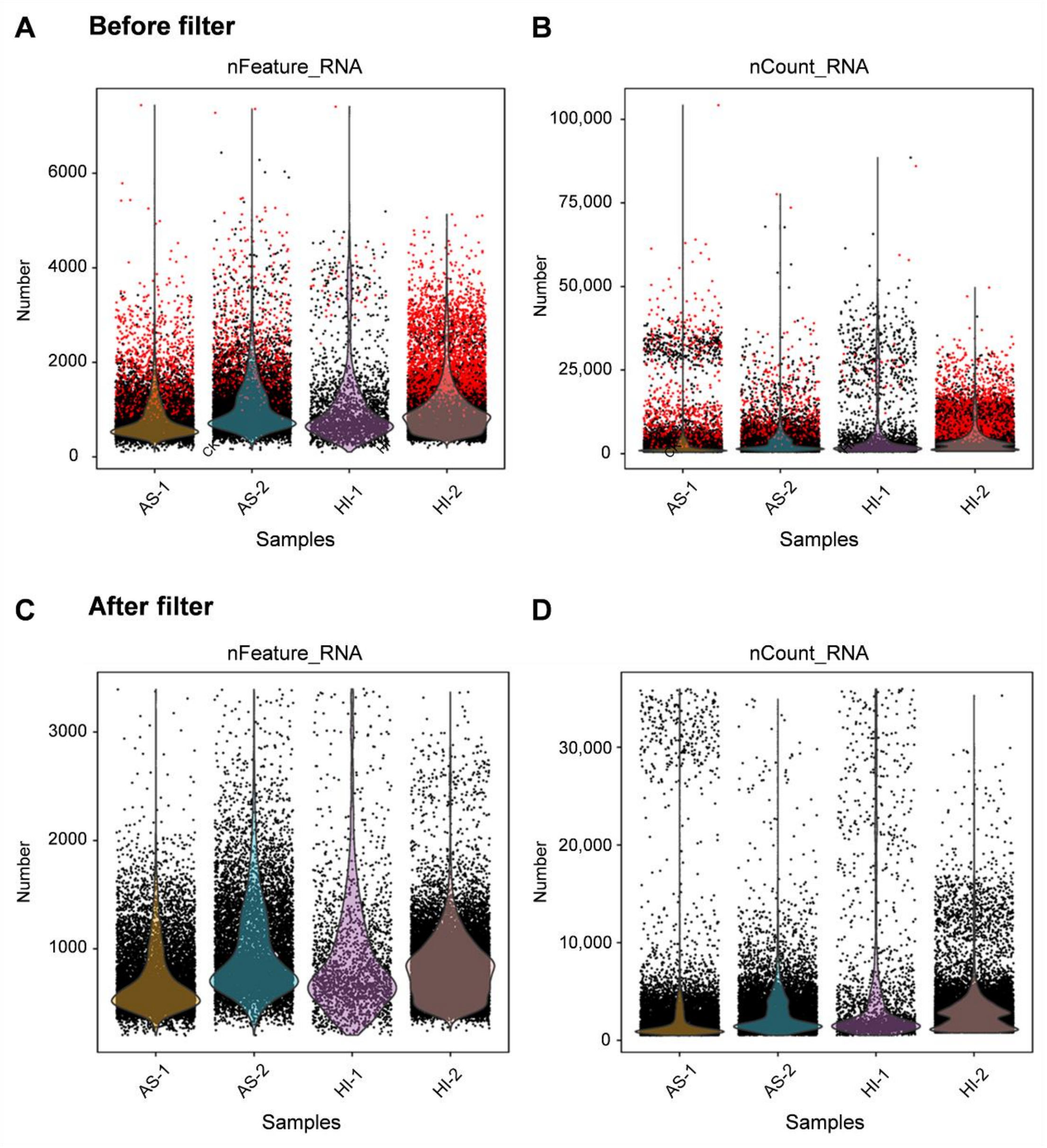

Figure S1. Information of scRNA-seq data quality control. (A) Cell number of *P. cantorii* hemocytes before quality control. (B). Count number of *P. cantorii* hemocytes before quality control. (C) Cell number of *P. cantorii* hemocytes after quality control. (D). Count number of *P. cantorii* hemocytes after quality control. NS and HI represent hemocytes of normal state and hibernation period, respectively.

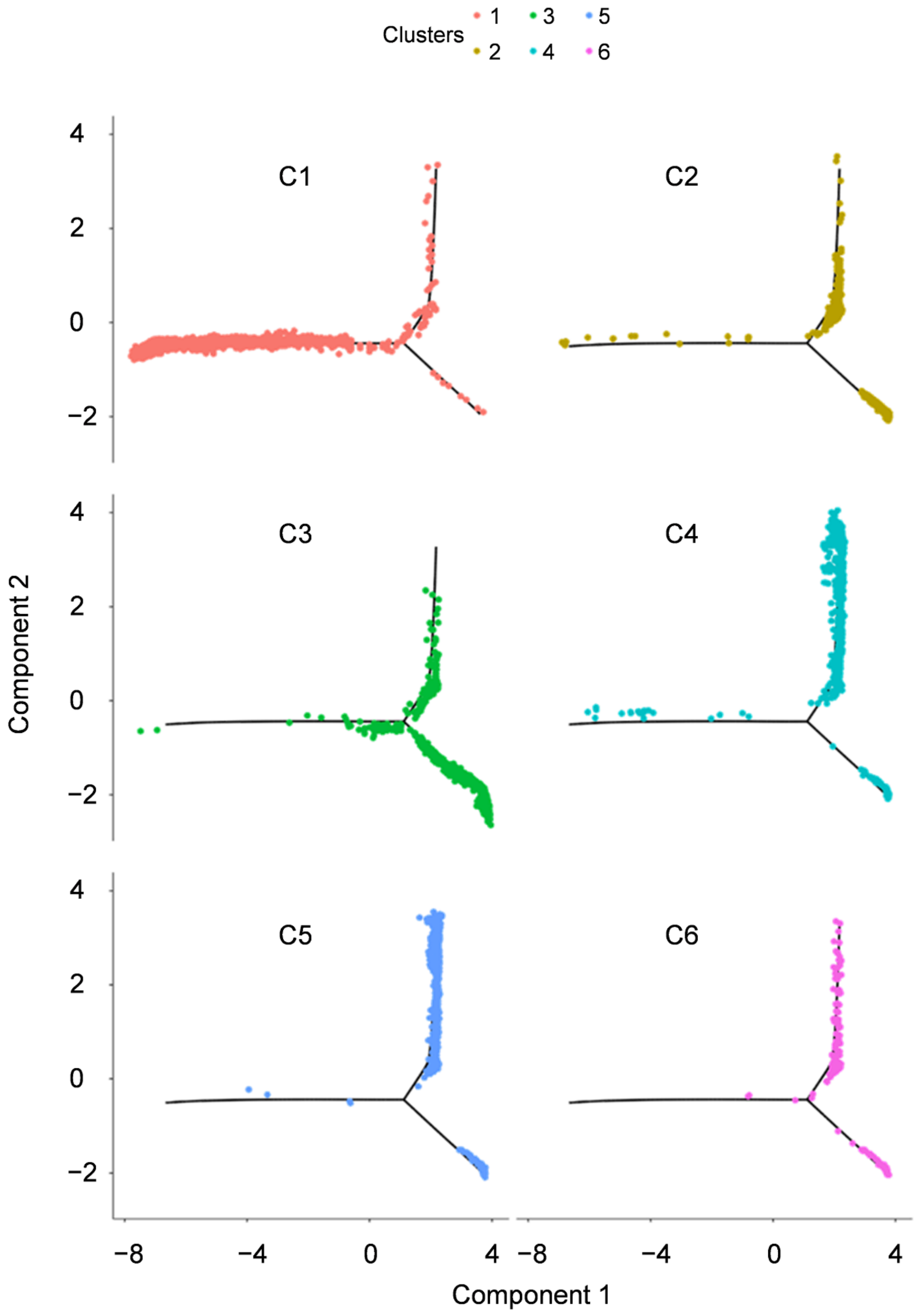

Figure S2. The subclusters of *P. cantorii* hemocytes distributed over the pseudo-time differentiation trajectory.

Table S1. Statistical analysis of cell data in each sample before and after filtration.

Sample	Before filtering number	After filtering number	Before filtering median UMI per cell	After filtering median UMI per cell	Before filtering median genes per cell	After filtering median genes per cell
AS	12,540	11,262	1,652	1,488	763	723
HI	13,547	11,104	2,507	2,251	827	765

Table S2. Statistical table of classification results of cell subsets

Cluster	AS	HI
C1	14691 (65.23%)	2180 (9.82%)
C2	140 (0.62%)	10285 (46.31%)
C3	1096 (4.87%)	6768 (30.48%)
C4	4972 (22.08%)	1516 (6.83%)
C5	1591 (7.06%)	1261 (5.68%)
C6	33 (0.15%)	198 (0.89%)

Table S4. The expression level of bipotent progenitor or stem-like cell signatures *zeb1* and *itga6* in hemocytes of normal state.

GeneID	Gene Name	C1	C2	C3	C4	C5	C6
Pca02G014240	*zeb1*	0.049	0.810	0.501	1.830	1.271	0.840
Pca07G003140	*itga6*	0.003	0.009	0.008	0.049	0.015	0.012
